# Cohort profile: The Singapore Breast Cancer Cohort (SGBCC), a multi-center breast cancer cohort for evaluation of phenotypic risk factors and genetic markers

**DOI:** 10.1371/journal.pone.0250102

**Published:** 2021-04-26

**Authors:** Peh Joo Ho, Yen Shing Yeoh, Hui Miao, Swee Ho Lim, Ern Yu Tan, Benita Kiat Tee Tan, Veronique Kiak Mien Tan, Su Ming Tan, Wei Sean Yong, Fuh Yong Wong, Preetha Madhukumar, Ching Wan Chan, Philip Tsau Choong Iau, Soo Chin Lee, Thomas Putti, Shaik Ahmad Buhari, Jin Yee Lee, Geok Hoon Lim, Evan Woo, Zhiyan Yan, Patrick Mun Yew Chan, Juliana Jia Chuan Chen, Sarah Qinghui Lu, Rebecca Dent, Wai Peng Lee, Chi Wei Mok, Jaime Chin Mui Seah, Xueling Sim, Rob Martinus van Dam, Kee Seng Chia, Jingmei Li, Mikael Hartman

**Affiliations:** 1 Saw Swee Hock School of Public Health, National University of Singapore and National University Health Systems, Singapore, Singapore; 2 Human Genetics, Genome Institute of Singapore, Singapore, Singapore; 3 Breast Department, KK Women’s and Children’s Hospital, Singapore, Singapore; 4 SingHealth Duke-NUS Breast Centre, Singapore, Singapore; 5 Department of General Surgery, Tan Tock Seng Hospital, Singapore, Singapore; 6 Division of Surgery and Surgical Oncology, National Cancer Centre Singapore, Singapore, Singapore; 7 Department of Breast Surgery, Singapore General Hospital, Singapore, Singapore; 8 Department of General Surgery, Changi General Hospital, Singapore, Singapore; 9 Division of Radiation Oncology, National Cancer Centre Singapore, Singapore, Singapore; 10 Department of Surgery, National University Hospital, Singapore, Singapore; 11 Division of Surgical Oncology, National University Cancer Institute, National University Health System, Singapore, Singapore; 12 Department of Oncology, Ng Teng Fong General Hospital, Singapore, Singapore; 13 Department of Haematology-Oncology, National University Cancer Institute, National University Health System, Singapore, Singapore; 14 Cancer Science Institute, National University of Singapore, Singapore, Singapore; 15 Department of Pathology, National University Hospital, Singapore, Singapore; 16 Department of General Surgery, Raffles Medical Group, Singapore, Singapore; 17 Division of Medical Oncology, National Cancer Centre Singapore, Singapore, Singapore; 18 Department of Surgery, Yong Loo Lin School of Medicine, National University of Singapore and National University Health Systems, Singapore, Singapore; Ohio State University Wexner Medical Center, UNITED STATES

## Abstract

This article aims to provide a detailed description of the Singapore Breast Cancer Cohort (SGBCC), an ongoing multi-ethnic cohort established with the overarching goal to identify genetic markers for breast cancer risk, prognosis and treatment response, as well as to understand the ethnic differences in disease risk and outcome in an Asian setting. The cohort comprises of breast cancer patients aged 21 years and above from six public hospitals which diagnose and treat nearly 76% breast cancer cases in Singapore. Self-reported data on sociodemographic and lifestyle, reproductive risk factors, medical history and family history of breast or ovarian cancer is collected using a structured questionnaire. Clinical data on tumour characteristics, and treatment modalities are obtained through medical record. Bio-specimens (blood or saliva) is collected at recruitment. Follow-up on survival information is done through routine linkage with the Registry of Births and Deaths. As of 31 December 2016, 7,768 subjects have been recruited to the study with 76% subjects contributed bio-specimens. The SGBCC provides a valuable platform which offers a unique, large and rich resource for new research ideas on breast cancer related phenotypic risk factors and genetic markers.

## Introduction

Global incidence rates of breast cancer are on the rise and more than two million women are diagnosed with the disease every year [1]. The increase in incidence can be largely attributable to a surge in breast cancer rates in Asia, possibly due to changes in lifestyle and reproductive profiles [2]. Recent studies have found that breast cancer rates in current Asian generations are surpassing even the historically high rates in the United States [3], highlighting an urgent need for efficient prevention and treatment strategies among Asian populations.

Aetiology, diagnosis, treatment and survivorship of breast cancer have been well-studied in western populations. Guidelines on breast cancer detection and treatment in many Asian countries are largely based on evidence from western studies. However, Asian women appear to be substantially different from European women in terms of lifestyles, reproductive profile, genetic susceptibility to breast cancer, cultural and religious beliefs related to health, socioeconomic status, and drug metabolism and response [4–8].

Singapore is a multi-ethnic country with three main ethnic groups, Chinese (75%), Malays (14%), and Indians (9%) [9]. It has attained one of the highest standards of living in Asia and established one of the most efficient healthcare systems in the world. In 2002, a population-based mammographic breast screening program—Breast Screen Singapore was established [10]. The participation rate under Breast Screen Singapore ranged from 9.9% to 13.7% for the reported periods 2002 to 2009 [10]. Age-standardized five-year relative survival rate of breast cancer has then improved from 50.2% in 1973–77 to 79.5% in 2008–2012, attributable to early detection and advances in cancer treatment in recent decades [11]. However, breast cancer is still the most common cancer and leading cause of cancer death among women in Singapore [12]. The breast cancer incidence rate in Singapore tripled from 23.8 per 100,000 in 1975–79 to 64.7 per 100,000 in 2010–2014, and is now amongst the highest in Asia [13]. A strong birth cohort effect was also observed, implying that gradual change towards a more westernized lifestyle has contributed to the increasing incidence rate, especially for the more recent birth cohorts [14]. The age-standardised incidence of breast cancer in Singapore is higher in Chinese women (66.0 per 100,000) compared to Malays (60.4 per 100,000) and Indians (58.8 per 100,000) [13]. Malay women have the worst five-year overall survival rate (58.5%) among the three ethnic groups as they are more likely to present at a younger age, with more advanced stages and more aggressive tumour biology [15]. The reasons for such ethnic differences remain unclear but it is possible that genetic, socioeconomic or cultural difference could play a role.

As such, the Singapore Breast Cancer Cohort Project (SGBCC) was established in 2010 to evaluate plausible genetic (germline) and non-genetic risk factors (e.g. lifestyle, demographic, reproductive, family history, etc.) pertaining to breast cancer. We aim to identify new biomarkers for prognosis and response to treatment, and understand the differences in survival among Asian ethnic populations through follow-up of the patients in the healthcare system or national registries. Finally, in collaboration with population-based women recruited from the Multi-Ethnic Cohort (MEC) [16], we can further identify new biomarkers for disease risk and diagnosis.

## Cohort description

### Study design and study population

SGBCC was first established as a cohort with both retrospective and prospective components. Recruitment started at National University Hospital (NUH, a tertiary academic hospital) in April 2010. Subsequently, recruitment sites extended to five other tertiary hospitals, namely KK Women’s and Children’s Hospital (KKH, in 2011), Tan Tock Seng hospital (TTSH, in 2013), National Cancer Centre Singapore (NCCS, in 2014), Singapore General Hospital (SGH, in 2015), and Changi General Hospital (CGH, in 2016) (S1 Fig). SGBCC was approved by the National Healthcare Group Domain Specific Review Board (reference number: 2009/00501) and the SingHealth Centralised Institutional Review Board (reference number: 2019/2246 [2010/632/B]).

At each participating site, eligible breast cancer patients are invited to participate during outpatient visits at breast surgeons’ or oncologists’ clinics. Eligibility is assessed with the following criteria: (1) a diagnosis of breast carcinoma in situ or invasive breast cancer; (2) citizens or permanent residents of Singapore; and (3) aged 21 years and above. Informed consent is sought by trained research coordinators in the patient’s language of choice (English, Chinese or Malay). In addition, information from medical records are stored at their respective hospitals, and are requested upon approval from institutional review board on project specific topics.

Consent for passive follow-up of participants is obtained. Disease outcomes such as recurrence, disease progression, and occurrence of other primary cancers are obtained from hospital medical records. Vital status and cause of death are obtained via linkage with the Registry of Births and Deaths in accordance to the audit cycle at each hospital [17]. In Singapore, nearly all deaths of citizens and permanent residents are certified. The certificate of cause of death is issued by doctors or authorized medical practitioners.

### Data collection

#### Structured questionnaire

The structured questionnaire was adapted from the KARolinska MAmmography Project for Risk Prediction of Breast Cancer (KARMA) study’s questionnaire and translated from the English version to Mandarin and Malay [18]. The questionnaire was self-administered in paper format, facilitated by a research coordinator. If the patient is illiterate, the research coordinator will read the questions to the participants in English, Mandarin or Malay.

Baseline information on sociodemographic factors are obtained at the time of recruitment. The variables include ethnicity, place of birth, marital status, employment status, type of housing, highest educational qualification attained, history of previous or existing illnesses such as diabetes, hypertension, and renal impairment, family history of breast cancer, family history of ovarian cancer, menstrual (age of menarche, age of menopause) and reproductive risk factors (parity status, age at first childbirth, breastfeeding) for breast cancer, use of oral contraceptives, use of hormonal replacement therapy, tobacco smoking, alcohol consumption, participation and attitudes towards mammographic screening program, and self-reported weight and height at time of recruitment. All variables and corresponding questions are available on https://blog.nus.edu.sg/sgbcc/for-researchers/.

#### Breast cancer registry and/or medical records

Hospitals have differing schedules in updating their in-house breast cancer registry, with collection of variables starting at different years. Where participants or variables are not found in the breast cancer registry, medical records are accessed from the electronic medical record system of the individual restructured hospitals in SGBCC. The electronic medical record system is widely adopted in government restructured hospitals in Singapore, with four of our sites (SGH, NCCS, KKH and CGH) sharing the same system. NUH and TTSH have independent electronic medical record systems. Variables are extracted two years after patients’ entry to the study to allow the maturation of treatment modalities and allow for quality checks by hospital staff. Extracted demographic variables include participant’s date of birth and date of diagnosis, tumour characteristics including tumour stage, tumour size (millimetre), tumour grade and histological type, estrogen receptor status, progesterone receptor status, human epidermal growth factor receptor 2 status, treatment-related variables including surgery, radiotherapy, adjuvant chemotherapy, neo-adjuvant chemotherapy, endocrine therapy, and targeted therapy.

Information on clinical variables are available at https://blog.nus.edu.sg/sgbcc/for-researchers/.

#### Biological specimens

Blood specimens of 20ml are obtained by the trained nurses or phlebotomists in the clinics after the interview or during subsequent clinical visits. Two types of blood tubes (BD Vacutainer^®^ Ethylenediaminetetraacetic acid (K2 EDTA) and BD Vacutainer^®^ Serum-separating tube (SST)) were used in all hospitals except for TTSH where two tubes of K2 EDTA were collected. Blood specimens are processed at a central biobank on a weekly basis. Upon receipt of the biological specimen, whole blood sample is separated into multiple aliquots of plasma, buffy coat, red blood cell, serum and blood clot after centrifugation. All aliquots are stored at temperature of -80 °C and an inventory tracking system is maintained to link with the biological specimen at all times. If the patient refuses donation of blood, a saliva specimen is collected by spitting into an Oragene^®^ DNA OG-500 self-collection kit manufactured by DNA Genotek^®^ [19, 20]. Saliva specimen in the original collection tube is stored at -80 °C before DNA extraction. DNA is extracted from buffy coat and saliva using Qiagen^®^ Flexigene DNA Kit (for buffy coat) and Oragene^®^ prepIT•L2P reagent (for saliva) according to the manufacturer’s protocol. Quantification of DNA is done using Trinean^®^ or NanoDrop^®^ platforms [21, 22]. DNA samples are stored in -20 °C for long-term storage.

#### Germline genetic information

**Fig 1:**
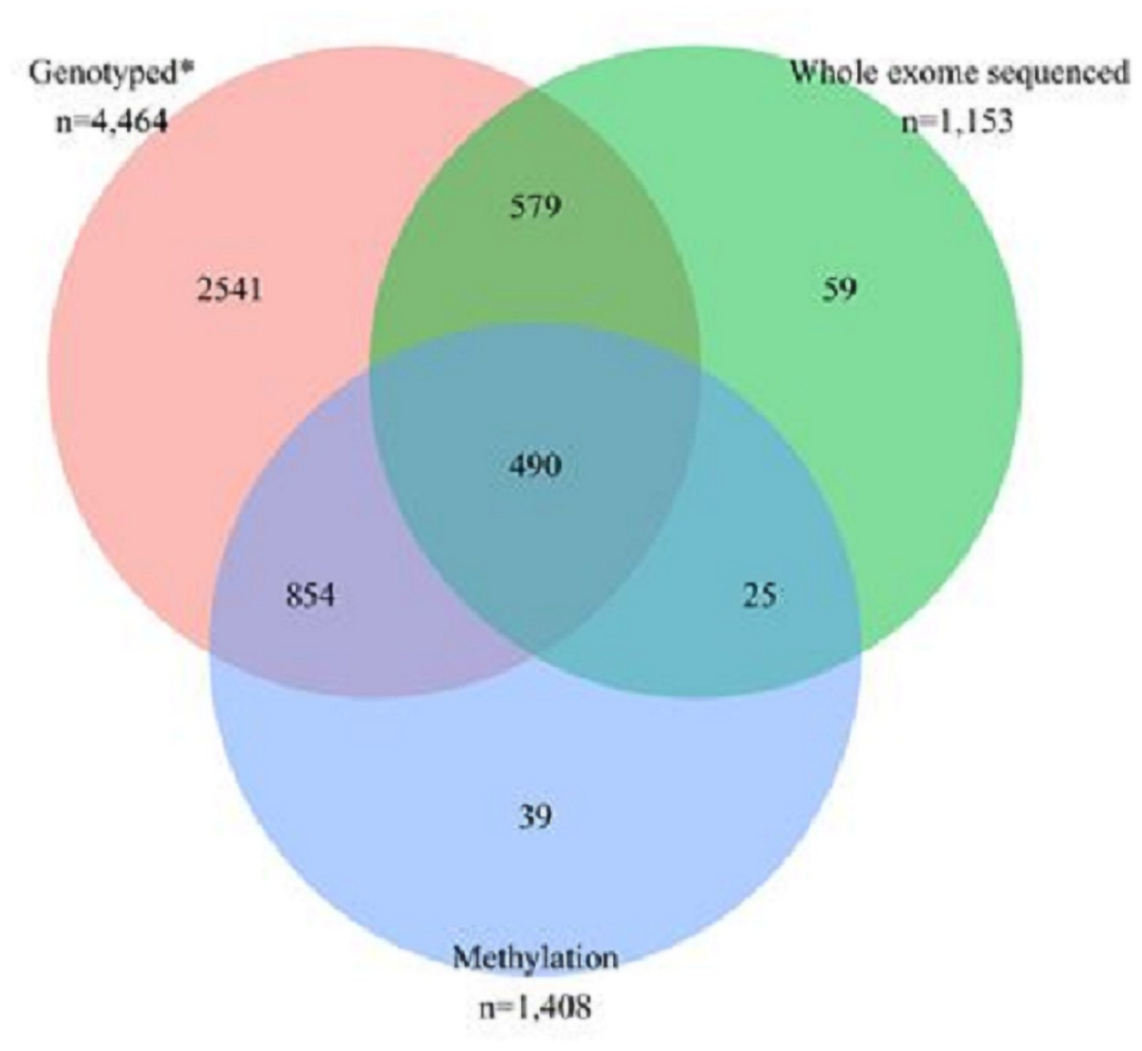
Participants who were genotyped using OncoArray, who were whole exome sequenced using Roche SeqCap EZ Human Exome v3.0, and/or had information on methylation levels measured using Illumina Infinium MethylationEPIC BeadChip. *All patients who were genotyped were part of Breast Cancer Risk after Diagnostic Gene Sequencing (BRIDGES).

In collaboration with the Breast Cancer Association Consortium (BCAC), 4,464 participants were genotyped using a custom single nucleotide polymorphism (SNP) genotyping array (illumina OncoArray-500K BeadChip) (Fig 1) [23]. The OncoArray contains ~500,000 SNPs with a genome- wide backbone of ~275,000 tag SNPs and additional content comprising variants associated with five common cancers (breast, colorectal, lung, ovarian, and prostate), ancestry, quantitative traits, and pharmacogenetics [24, 25]. As part of Breast Cancer Risk after Diagnostic Gene Sequencing (BRIDGES) initiative [26], next-generation targeted sequencing of 34 genes known or are suspected to be associated with breast cancer was performed for 4,464 patients (Fig 1) [27]. Of the 4,464 patients, 385 have at least one protein truncating variants in any of the 34 genes studied (S2 Fig). In addition, whole-exome sequencing (Roche SeqCap EZ Human Exome v3.0) and array-based profiling of DNA methylation (Illumina Infinium MethylationEPIC BeadChip) was also performed for a subset of 1,153 and 1,408 breast cancer patients, respectively.

## Findings to date

**Fig 2:**
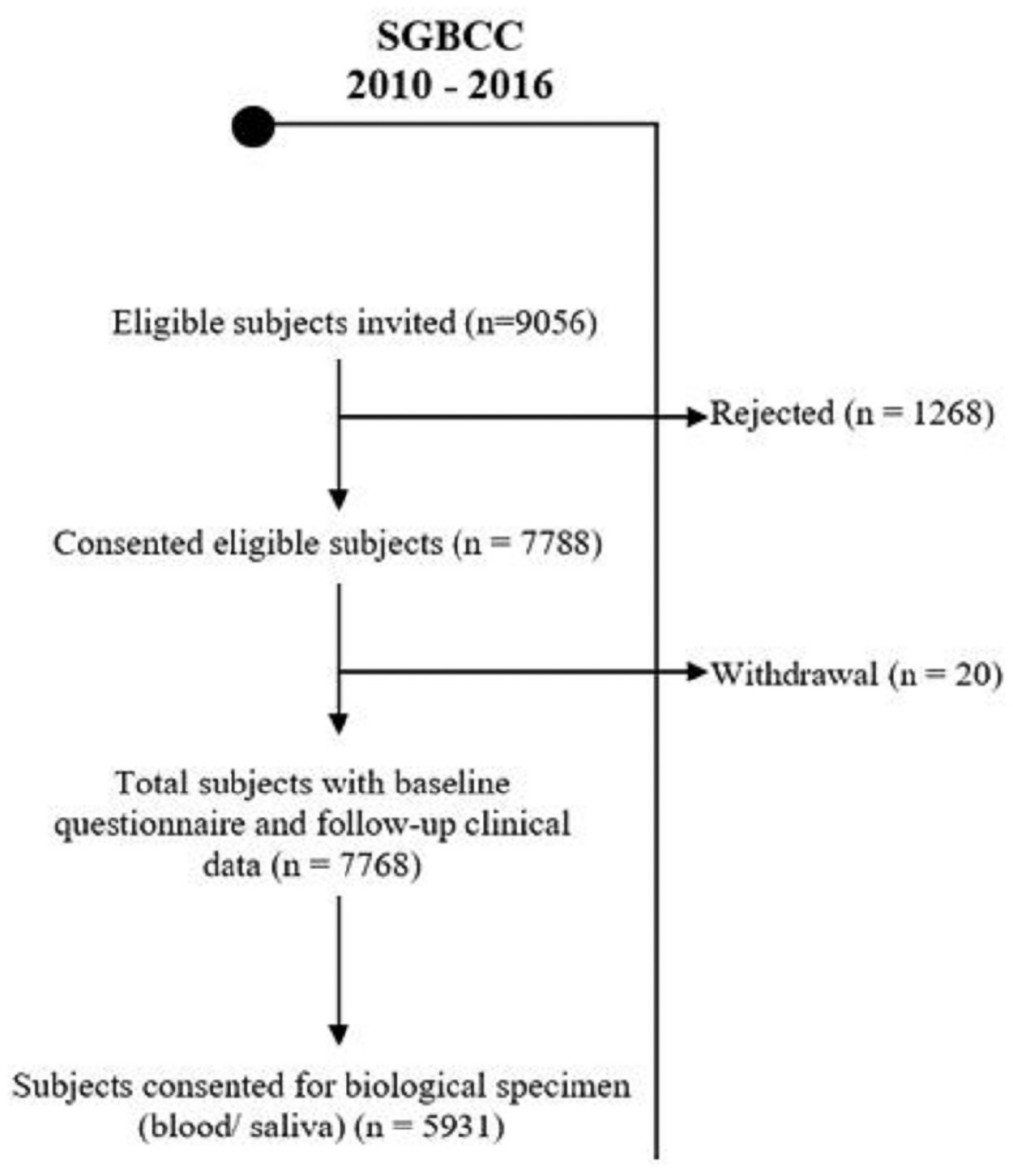
Flow chart of study participants as of 31 December 2016.

The recruitment for SGBCC is still ongoing. As of 31 December 2016, 7,768 breast cancer patients have been enrolled and full clinical data is available. The overall participation rate was 86% and 5,931 (76%) subjects contributed bio-specimens (Fig 2). The cohort grew quickly in the first two years of recruitment at each participating site, mainly driven by prevalent cases (recruitment was more than one year post diagnosis of breast cancer) of participants on routine surveillance, and then slowed down to a steady rate of 200 newly diagnosed patients per year thereafter.

**Fig 3:**
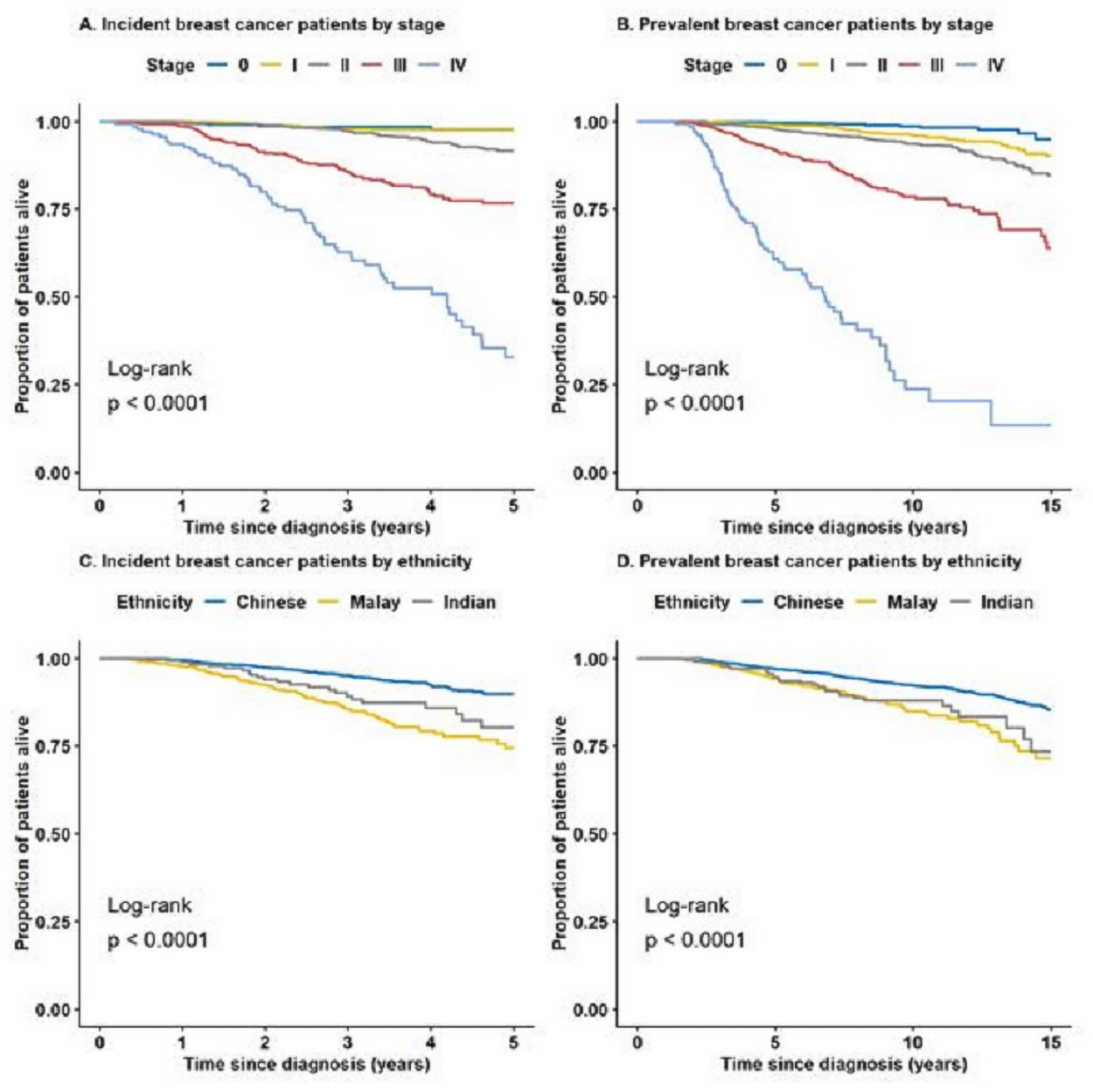
Overall survival of breast cancer patients by stratified case type (incident, prevalent) and stage at diagnosis (A and B), or ethnicity (C and D).

Table 1 shows the comparisons between participant characteristics and national-level statistics reported in the Singapore cancer registry report on female breast cancer in 2010–2014 [13], Singapore census in 2010 [9], and National Health Survey in 2010 [28] (Table 1). The associations between selected clinical characteristics and case status (incident or prevalent breast cancer), using the Chi-square test are summarized in Table 2. A total of 626 deaths, 458 due to breast cancer, was observed. Difference (log rank p-value<0.0001) in overall survival was observed between the stages at diagnosis and between the three major ethnicities (Fig 3). The observed survival difference by ethnicity is in agreement with the existing literature reporting that certain ethnic groups, such as Malays, are independently associated with worse survival [15]. Further studies are needed to clarify the underlying reasons.

**Table 1:** Body mass index, lifestyle and demographic profile of SGBCC participants, recruited between April 2010 and Dec 2016, compared with the general population in Singapore.

	SGBCC		General population %	Difference
	n	%		
**Age at diagnosis** ^a^				
0–44	1,593	20.5	17.9	2.6
45–54	2,725	35.1	29.0	6.1
55–64	2,255	29.0	29.1	-0.1
65–74	921	11.9	14.8	-2.9
75+	272	3.5	9.2	-5.7
Unknown	2	0.0		
**Ethnicity** ^a^				
Chinese	6,142	79.1	80.5	-1.4
Malay	925	11.9	10.6	1.3
Indian	448	5.8	6.3	-0.5
Others	253	3.3	2.5	0.8
**Highest educational qualification attained (female, aged 15+)** ^b^				
Below secondary	2,308	29.7	35.5	-5.8
Secondary	2,879	37.1	19.9	17.2
Post-secondary ^c^	2,563	33.0	44.6	-11.6
Unknown	18	0.2		
**Housing type** ^b^				
1–3 room HDB flat	1,737	22.4	24.6	-2.2
4 room HDB flat	2,627	33.8	31.9	1.9
5 room and above HDB flat	2,223	28.6	25.9	2.7
Condominium and private flats	696	9.0	11.5	-2.5
Landed property and others	481	6.2	6.1	0.1
Unknown	4	0.1		
**Mammography screening ever (female, aged 50 to 69 years)** ^d^				
Yes	2,248	45.1	66.3	-21.2
No	1,486	29.8	33.7	-3.9
Unknown	1,253	25.1		
**Number of children (ever married)** ^e^				
0	602	9.0	11.8	-2.8
1	1,054	15.8	19.2	-3.4
2	2,634	39.4	34.4	5.0
≥3	2,395	35.8	34.6	1.2
Unknown	7	0.1		
**Smoking status** ^f^				
Non-smoker	7,569	97.4	92.5	4.9
Smoker	193	2.5	7.5	-5.0
Unknown	6	0.1		
**Body mass index (kg/m2)** ^f^				
Underweight (<18.5)	1,098	14.1	8.2	5.9
Normal (18.5–24.9)	3,576	46.0	58.0	-12.0
Overweight (≥25)	2,999	38.6	33.8	4.8
Unknown	95	1.2		

^a^ Singapore Cancer Registry (female breast).

^b^ Singapore Census of Population 2010.

^c^ Post-secondary (non-tertiary), Diploma and Professional Qualification, and University.

^d^ National Health Survey 2010 (female, aged 50 to 69 years).

^e^ https://www.singstat.gov.sg/publications/population/census10_admin.

^f^ https://www.moh.gov.sg/resources-statistics/reports/national-health-survey-2010.

**Table 2:** Association between clinical characteristics and case status (incident or prevalent breast cancer), using the Chi-square test.

	Total N = 7,768	Case-type		P-value ^a^
		Incident N = 3,316	Prevalent N = 4,452	
**Tumour characteristics**				
**Tumour behaviour**				
In-situ	1,113 (14)	406 (12)	707 (16)	0.012
Invasive	5,891 (76)	2,388 (72)	3,503 (79)	
Unknown	764 (10)	522 (16)	242 (5)	
**Tumour histology type**				
Ductal	6,133 (79)	2,390 (72)	3,743 (84)	<0.001
Lobular	304 (4)	137 (4)	167 (4)	
Mucinous	211 (3)	90 (3)	121 (3)	
Others	356 (5)	177 (5)	179 (4)	
Unknown	764 (10)	522 (16)	242 (5)	
**Nodal status**				
Negative	4,763 (61)	1,973 (59)	2,790 (63)	<0.001
Positive	2,577 (33)	1,176 (35)	1,401 (31)	
Unknown	428 (6)	167 (5)	261 (6)	
**Tumour size**				
T0	1,108 (14)	411 (12)	697 (16)	<0.001
T1	2,932 (38)	1,137 (34)	1,795 (40)	
T2	2,535 (33)	1,185 (36)	1,350 (30)	
T3	406 (5)	198 (6)	208 (5)	
T4	307 (4)	164 (5)	143 (3)	
Unknown	480 (6)	221 (7)	259 (6)	
**Tumour stage**				
0	1,111 (14)	411 (12)	700 (16)	<0.001
I	2,182 (28)	847 (26)	1,335 (30)	
II	2,678 (34)	1,212 (37)	1,466 (33)	
III	1,163 (15)	542 (16)	621 (14)	
IV	272 (4)	141 (4)	131 (3)	
Unknown	362 (5)	163 (5)	199 (4)	
**Tumour grade**				
Well differentiated	1,174 (15)	446 (13)	728 (16)	0.005
Moderate differentiated	2,788 (36)	1,170 (35)	1,618 (36)	
Poor differentiated	2,858 (37)	1,246 (38)	1,612 (36)	
Unknown	948 (12)	454 (14)	494 (11)	
**Estrogen receptor**				
Positive	5,005 (64)	2,271 (68)	2,734 (61)	0.392
Negative	1,549 (20)	683 (21)	866 (19)	
Unknown	1,214 (16)	362 (11)	852 (19)	
**Progesterone receptor**				
Positive	4,411 (57)	2,012 (61)	2,399 (54)	0.051
Negative	2,084 (27)	896 (27)	1,188 (27)	
Unknown	1,273 (16)	408 (12)	865 (19)	
**Human epidermal growth factor receptor 2**				
Positive	1,504 (19)	761 (23)	743 (17)	0.009
Negative	3,797 (49)	1,768 (53)	2,029 (46)	
Unknown	2,467 (32)	787 (24)	1,680 (38)	
**Treatment**				
**Breast surgery**				
Yes	7,132 (92)	3,089 (93)	4,043 (91)	0.006
No	166 (2)	90 (3)	76 (2)	
Unknown	470 (6)	137 (4)	333 (7)	
**Radiotherapy**				
Yes	4,038 (52)	1,702 (51)	2,336 (52)	0.015
No	2,877 (37)	1,298 (39)	1,579 (35)	
Unknown	853 (11)	316 (10)	537 (12)	
**Neo-adjuvant chemotherapy**				
Yes	721 (9)	440 (13)	281 (6)	<0.001
No	5,066 (65)	1,984 (60)	3,082 (69)	
Unknown	1,981 (26)	892 (27)	1,089 (24)	
**Adjuvant chemotherapy**				
Yes	3,861 (50)	1,551 (47)	2,310 (52)	0.069
No	2,489 (32)	1,058 (32)	1,431 (32)	
Unknown	1,418 (18)	707 (21)	711 (16)	
**Endocrine therapy**				
Yes	4,604 (59)	1,863 (56)	2,741 (62)	0.577
No	1,790 (23)	710 (21)	1,080 (24)	
Unknown	1,374 (18)	743 (22)	631 (14)	
**Targeted therapy**				
Yes	621 (8)	271 (8)	350 (8)	0.072
No	4,031 (52)	1,602 (48)	2,429 (55)	
Unknown	3,116 (40)	1,443 (44)	1,673 (38)	

Column percentages are presented.

^a^ Differences between incidence and prevalence cases were assessed using chi square testing.

**Fig 4:**
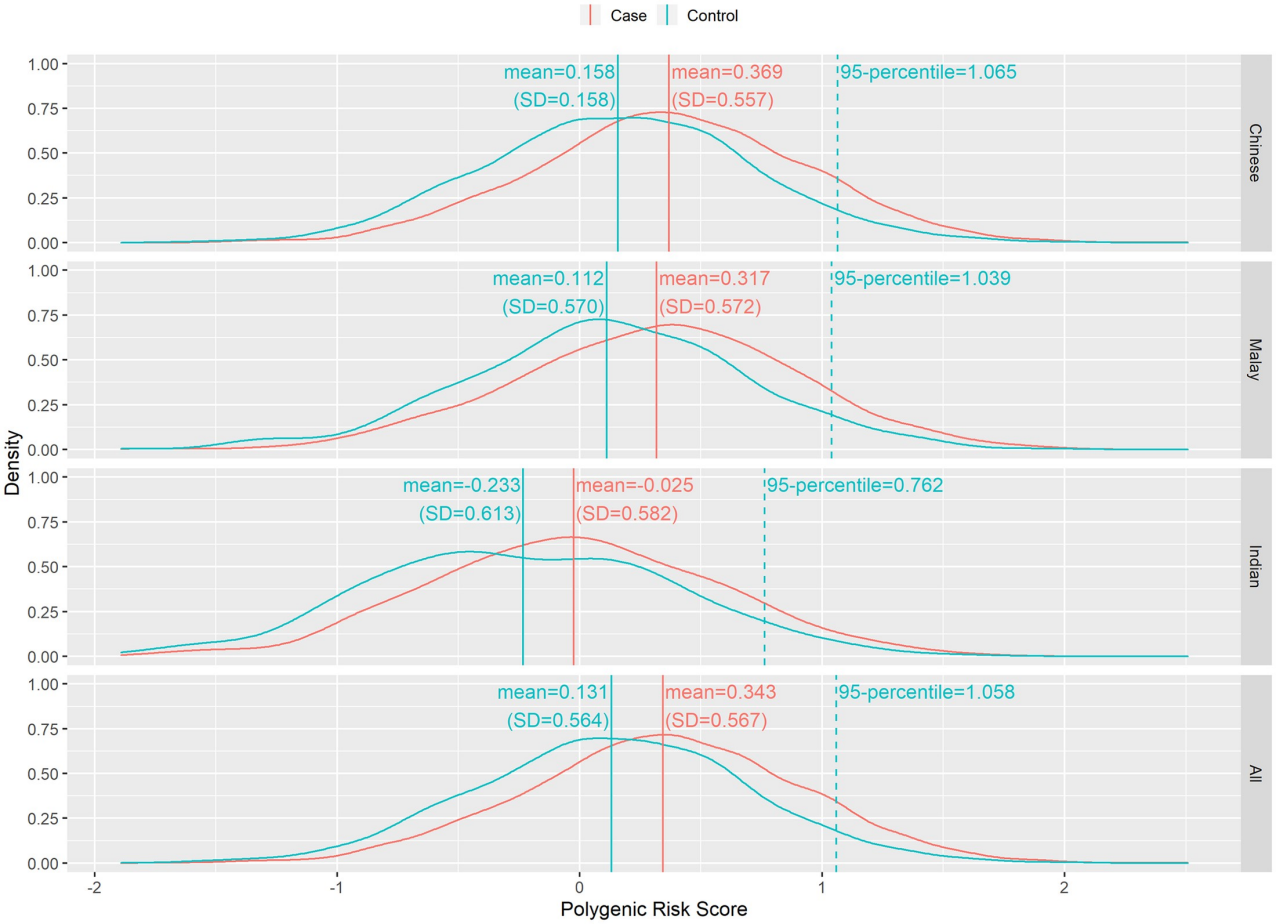
Distribution of polygenic risk scores for cases and controls by ethnicity groups.

S1 Table lists the protein truncating variants (PTVs) carriership for breast cancer patients from SGBCC. We used the Singapore MEC as controls to compare the frequency of PTVs in the three major ethnic groups (Chinese, Malay and Indian) [16]. The MEC enrolled 34,870 males and females from the general population between 2013 and 2016 and aims to monitor risk factors on development of common health conditions (http://blog.nus.edu.sg/sphs/multiethnic-cohort/) [16]. We matched 4,124 controls from MEC to 4,457 breast cancer patients by ethnicity and age (enrolment age of +/- 5 years from the age at diagnosis of patients). S2 Table presents the frequency of rare protein truncating variants (PTVs) carriership for breast cancer patients from SGBCC and controls from MEC by ethnicity. To date, the most robust and reliable breast cancer risk predictor comprised of common genetic variants identified by GWAS is the 313-SNP breast cancer polygenic risk score developed based on women of European ancestry [29]. Genotyping was done for controls from MEC with matched cases from SGBCC. The distribution of PRS differed for cases and controls for the different ethnic groups (Kruskal-Wallis test p-value, P_Chinese_ = 1.79E-49, P_Malay_ = 7.70E-12, and P_Indian_ = 4.44E-7) (Fig 4).

SGBCC contributed to BCAC and the Asian Breast Cancer Consortium, S3 Table presents three of the recent works where SGBCC had notable contributions [26, 30, 31]. A full list of publications generated by the cohort can be found here https://blog.nus.edu.sg/sgbcc/publications/.

## Strengths and limitations

The SGBCC is one of the largest breast cancer cohort studies in Asia and has several unique strengths. Annually, approximately 1,800 women in Singapore are diagnosed with breast cancer and reported to Singapore Cancer Registry. SGBCC recruits breast cancer patients from six of the ten restructured hospitals in Singapore; Three of the restructured hospitals not part of SGBCC were established in 2010 (Khoo Teck Puat Hospital), 2015 (Ng Teng Fong General Hospital) and 2018 (Sengkang General Hospital). Our cohort participants were of similar distribution of age and ethnicity to breast cancer patients in Singapore as identified by the Singapore Cancer Registry, which strengthened the generalisability of the study. The participation rate of the current study was high (86%) across all study sites, of which 76% of our cohort participants have donated saliva or blood sample. With high-level adoption of electronic medical record among healthcare institutions in Singapore, additional direct patient contact is not required for follow-up. Ensuring a higher participation rate, higher accuracy of clinical information and reducing the potential of loss to follow-up.

We acknowledge that there are some limitations to our cohort. As with all prevalent case cohort studies, survivorship bias is observed. Only patients that were alive at the time of enrolment were included and patients diagnosed in years prior to the start of recruitment would tend to be of better prognosis. Patients with short survival time and low compliance to post-treatment surveillance and follow-up clinical care were more likely to be missed. Thus, the cohort on prevalent cases is biased towards more favourable survival outcome. However, the proportion of incidence cases at each site increases to approximately 80% after a period of five years. In addition, a study has demonstrated that the inclusion of prevalent cases in a population-based epidemiological cohort of breast cancer patients does not bias the hazard ratio estimation for three prognostic factors—clinical stage, grade and estrogen receptor status in a left truncation Cox survival analysis when the proportional hazards assumption holds [32]. Information on exposure variables prior to the occurrence of breast cancer like menstrual and reproductive risk factors may be subjected to recall bias. Socially desirability response bias to questions on tobacco smoking, alcohol consumption, physical activities, and participation and attitudes towards mammographic screening program may occur.

## Supporting information

S1 Fig Subject recruitment chart of the Singapore Breast Cancer Cohort (SGBCC) study. Breast cancer patients are approached by trained research coordinators during their outpatient visit at SGBCC hospital sites. Informed consent is sought in the patient’s language of choice (English, Chinese or Malay). Over an in-person interview with a research coordinator, participants answer a comprehensive questionnaire for assessing known breast cancer risk factors and attitude towards mammography screening. A blood or saliva sample was taken. Information on tumor characteristics, treatment, recurrence, survival and other adverse outcomes are retrieved from medical records. Date and cause of death are updated via record linkage to a national registry.
(PDF)

S2 Fig Oncoplot of 4464 patients with breast cancer with at least one rare protein truncating variant (PTV) in any of the 34 genes studied. No PTV was found in *AKT1*, *BABAM2*, *CDH1*, *MEN1*, *MLH1*, *NBN*, *PIK3CA*, *and STK11*. Each column represents one patient.
(PDF)

S1 Table List of predicted protein-truncating variants in 4,464 breast cancer patients from Singapore Breast Cancer Cohort. (XLSX)

S2 Table Frequency of protein truncating variables carriership of the 34 genes tested by ethnicity (Chinese, Malay, and Indian). Cases from Singapore Breast Cancer Cohort and controls from the Multi-ethnic Cohort of Singapore were sequenced together. Neither cases nor controls carried protein truncating variants in seven genes (*ABRAXIS1*, *BABAM2*, *CDH1*, *MEN1*, *MLH1*, *MSH2*, *PIK3C*, *and STK11*).
(XLSX)

S3 Table Notable studies that SGBCC has contributed to. (XLSX)
