# Supplementary material for: Cohort profile: The Singapore Breast Cancer Cohort (SGBCC), a multi-center breast cancer cohort for evaluation of phenotypic risk factors and genetic markers
Source: PLoS One. 2021 Apr 26;16(4):e0250102. doi: 10.1371/journal.pone.0250102 (PMC8075208; doi:10.1371/journal.pone.0250102)

**S1 Fig**: Subject recruitment chart of the Singapore Breast Cancer Cohort (SGBCC) study. Breast cancer patients are approached by trained research coordinators during their outpatient visit at SGBCC hospital sites. Informed consent is sought in the patient's language of choice (English, Chinese or Malay). Over an in-person interview with a research co-ordinator, participants answer a comprehensive questionnaire for assessing known breast cancer risk factors and attitude towards mammography screening. A blood or saliva sample was taken. Information on tumor characteristics, treatment, recurrence, survival and other adverse outcomes are retrieved from medical records. Date and cause of death are updated via record linkage to a national registry.

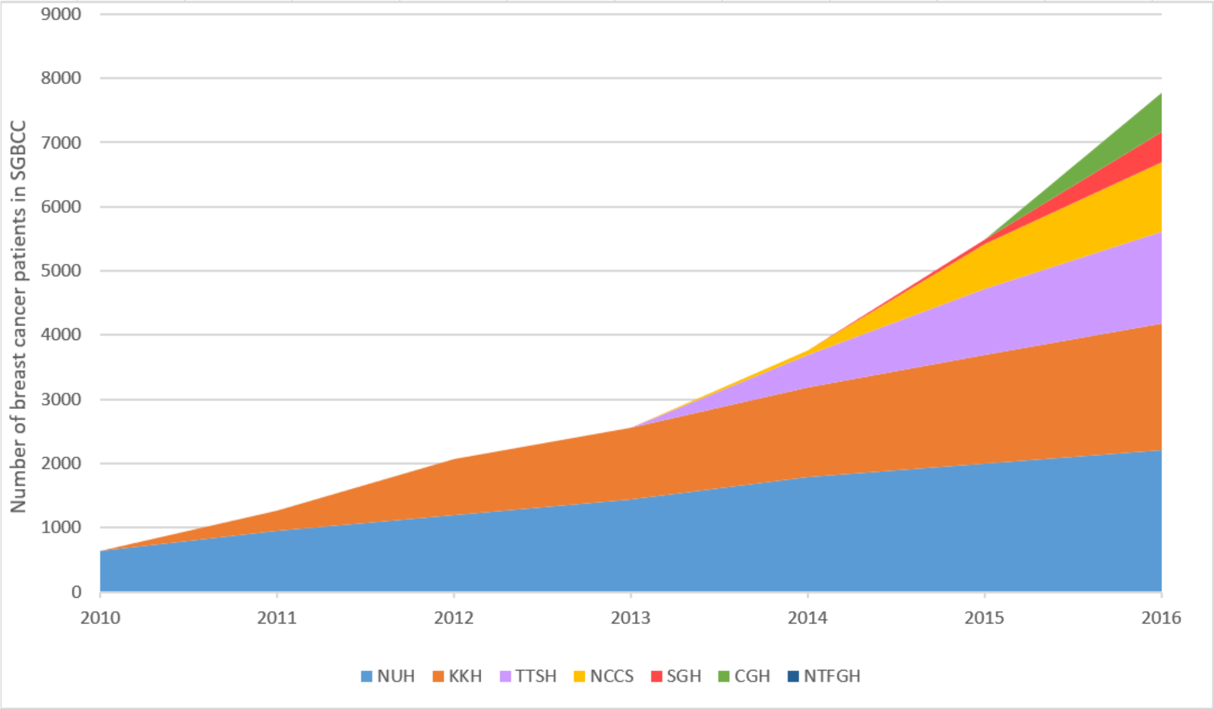

Supplement: S1 Fig — Breast cancer patients are approached by trained research coordinators during their outpatient visit at SGBCC hospital sites. Informed consent is sought in the patient’s language of choice (English, Chinese or Malay). Over an in-person interview with a research coordinator, participants answer a comprehensive questionnaire for assessing known breast cancer risk factors and attitude towards mammography screening. A blood or saliva sample was taken. Information on tumor characteristics, treatment, recurrence, survival and other adverse outcomes are retrieved from medical records. Date and cause of death are updated via record linkage to a national registry. (PDF) [file pone.0250102.s001.pdf]
