# Supplementary material for: Cohort profile: The Singapore Breast Cancer Cohort (SGBCC), a multi-center breast cancer cohort for evaluation of phenotypic risk factors and genetic markers
Source: PLoS One. 2021 Apr 26;16(4):e0250102. doi: 10.1371/journal.pone.0250102 (PMC8075208; doi:10.1371/journal.pone.0250102)

S2 Fig. Oncoplot of 4464 patients with breast cancer with at least one rare protein truncating variant (PTV) in any of the 34 genes studied. No PTV was found in AKT1, BABAM2, CDH1, MEN1, MLH1, NBN, PIK3CA, and STK11. Each column represents one patient.

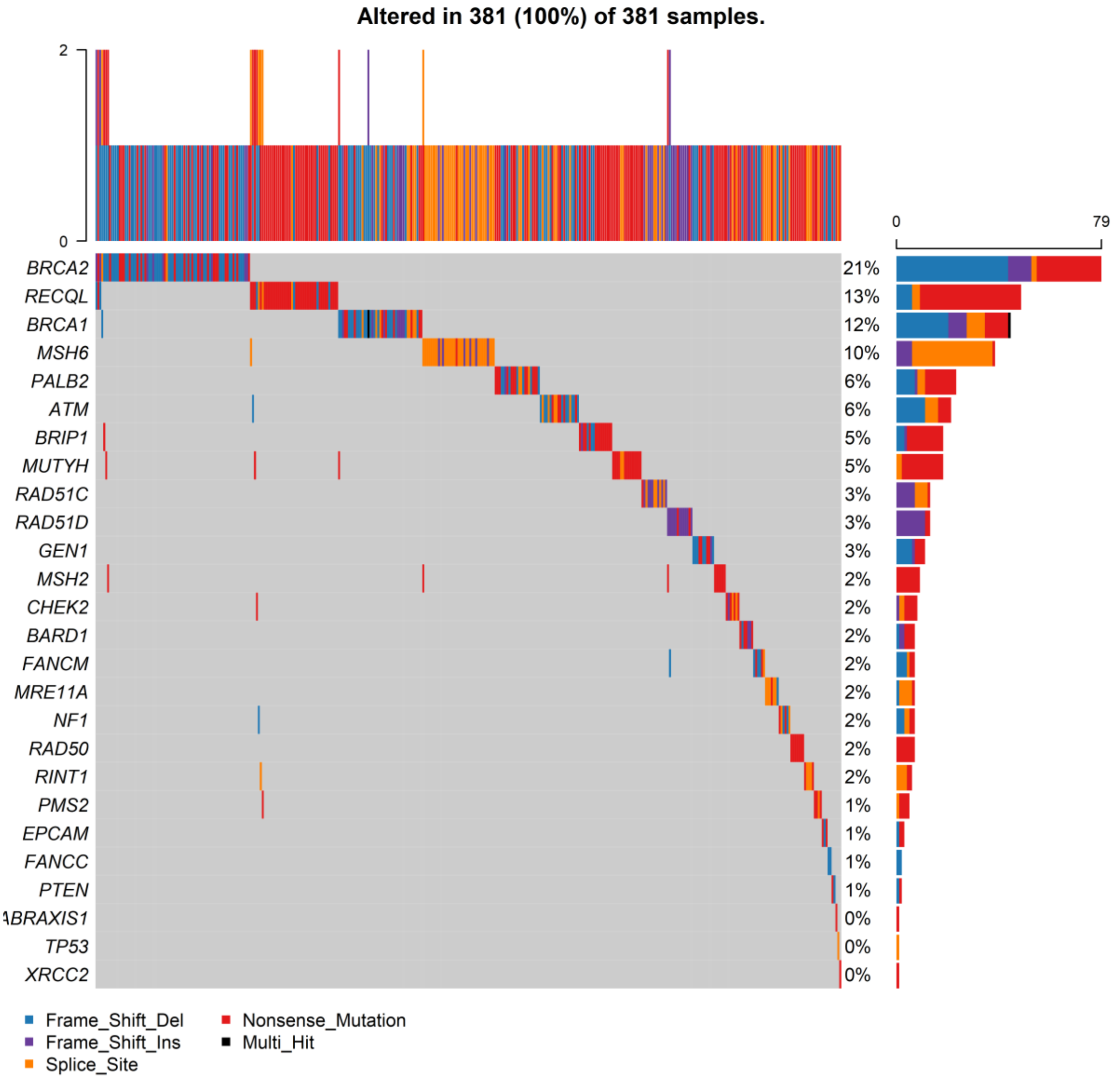

Supplement: S2 Fig — No PTV was found in AKT1, BABAM2, CDH1, MEN1, MLH1, NBN, PIK3CA, and STK11. Each column represents one patient. (PDF) [file pone.0250102.s002.pdf]
